# Plasma and Urine Pharmacokinetics of Long‐Acting Injectable Omeprazole Following Intramuscular Administrations to Healthy Thoroughbred Horses

**DOI:** 10.1111/jvp.13494

**Published:** 2025-02-08

**Authors:** Caitlin Harding, Marjaana Viljanto, Pamela Hincks, Jocelyn Habershon‐Butcher, Stuart W. Paine

**Affiliations:** ^1^ LGC Limited Fordham UK; ^2^ British Horse Racing Authority London UK; ^3^ School of Veterinary Medicine and Science University of Nottingham Sutton Bonington UK

**Keywords:** detection time, horse, intramuscular, omeprazole, pharmacokinetics

## Abstract

Omeprazole is a gastric acid secretion inhibitor used as an effective anti‐ulcer drug. Based on oral administration studies, its International Screening Limit (ISL) was established in plasma and urine at 1 ng/mL with a Detection Time (DT) of 48 h. A novel formulation of injectable omeprazole has since been released, and therefore, a pharmacokinetic study was performed to assess the DT above the ISL against current advice. Six Thoroughbred horses were given four repeated weekly intramuscular administrations of omeprazole (4 mg/kg). Plasma and urine omeprazole concentrations were measured by liquid chromatography–tandem mass spectrometry. Based on the current plasma and urine ISL (1 ng/mL), the DT for this long‐acting omeprazole formulation administered at 4 mg/kg once per week is greater than 384 h (16 days) in both plasma and urine. Thus realistically, despite the appeal of giving an injection once per week rather than oral medication daily over a long period of time, this would make treatment for horses in training with the long‐acting product challenging within the rules of racing. It would therefore most likely be used for horses outside of training, and the oral formulation would still be legitimately used during training.

## 1 Introduction

Omeprazole, a substituted benzimidazole, is a proton pump inhibitor that reduces gastric acid secretion and is used as an effective anti‐ulcer drug. In the horse, it is used orally to treat equine squamous gastric disease (ESGD) and equine glandular gastric disease (EGGD), which are subcategories of equine gastric ulcer syndrome (EGUS) dependent on the region of the stomach affected by ulcerative lesions (Sykes, Hewetsone, et al. 2015). ESGD is prevalent amongst all populations of horses, with Thoroughbred horses most affected. There is a general increase in occurrences during high‐intensity training or competitive periods, with 80%–100% of Thoroughbred horses affected within 2–3 months of race training (Begg and O'Sullivan 2003; Murray et al. 1996; Sykes, Hewetsone, et al. 2015; Vatistas et al. 1999).

As a part of medication control by the regulators, horses are not allowed to race under the pharmacological effects of drugs but should be treated appropriately in training for health and welfare purposes. Therefore, to control for the use of omeprazole on race day while still allowing for the treatment of EGUS in susceptible horses during the lead‐up to the event, determining the therapeutic concentrations and withdrawal time is necessary. The European Horserace Scientific Liaison Committee (EHSLC) is a technical group representing European racing regulatory authorities who develop harmonized medication control advice based on detection times (DTs; the time between the last time a drug was administered and when the measured drug's concentration in a horse's system is low enough to have no significant effect) and associated international screening limits (ISLs; the detection limits to be used by the laboratories when screening for certain therapeutic substances as instructed by the authorities). Previous pharmacokinetic studies, adhering to EHSLC guidelines, have been carried out for various oral formulations of omeprazole, including GastroGard paste (Hannan et al. 2008) and UlcerGold (Viljanto et al. 2018). Based on evidence from these studies, the current ISL of 1 ng/mL and DT of 48 h were established for both plasma and unhydrolyzed urine (International Federation of Horseracing Authorities 2021a, 2021b) to enable veterinarians to determine when the horses may be raced following treatment.

Oral omeprazole was authorized for the treatment and prevention of EGUS in the United States in 1999 and shortly thereafter in other countries (Gough, Hallowell, and Rendle 2022). However, whilst the healing of ESGD is relatively high with this treatment (67%–94%), more recent work has indicated limited healing of EGGD (9%–50%) with oral omeprazole use alone (Gough, Hallowell, and Rendle 2022, 2020; Sykes, Sykes, and Hallowell 2014). Long‐acting injectable omeprazole is a relatively novel suspension formulation and uses an intramuscular (IM) administration route. The required administration intervals are longer when using the injectable substance (once a week) compared to the oral products (once a day), which may prove more convenient and thus improve treatment compliance. Also, this administration route bypasses the stomach, so it should avoid the variation in bioavailability that has been reported with oral formulations (Lehman et al. 2021; Sykes et al. 2017). Furthermore, recent studies have evidenced that treatment with long‐acting injectable omeprazole resulted in higher rates of ESGD (97%–100%) and EGGD healing (52%–75%) when compared to oral omeprazole formulations (Gough, Hallowell, and Rendle 2022, 2020; Sykes et al. 2017). Treatment failure rates were greatly reduced when using long‐acting injectable omeprazole when compared to oral formulations (3% versus 33% for ESGD and 18% versus 50% for EGGD) (Gough, Hallowell, and Rendle 2022, 2020).

Considering the benefits offered by long‐acting injectable omeprazole, it is expected that it will be licensed for veterinary use. Currently the UK product (BOVA) is used by veterinarians in compliance with the veterinary prescribing cascade (Veterinary Medicines Directorate (VMD)), Cascade‐ extemporaneous preparations, specials [VMD], and the RCVS code of professional conduct for veterinarians. To allow for its control in a regulatory setting, information on the DT and associated ISL following administration must be gathered to develop harmonized medication control advice related to the novel formulation. The aims of this six‐horse study were to assess pharmacokinetics following IM administration of long‐acting injectable omeprazole (BOVA UK), adhering to EHSLC guidelines, and to determine whether the established ISL and DT of omeprazole in unhydrolyzed urine and plasma are suitable with this novel administration route.

## 2 Materials and Methods

### 2.1 Drug Administration and Sampling

Six healthy Thoroughbred horses (four geldings, two mares/fillies), aged 3–5 years old with a mean ± SD body weight of 474 ± 46 kg, were administered injectable omeprazole to the gluteal muscle (BOVA UK; Greater London, UK). Horses received 4 mg/kg IM doses of omeprazole once a week for 4 weeks (dose times recorded at approximately −504, −336, −168 and 0 h). They were exercised in a manner consistent with that used in British training yards, fed solely a normal racehorse diet, and housed at the British Horseracing Authority's Centre for Racehorse Studies (CRS; Suffolk, UK). All horses were at least 1 month without medications prior to the study. Ethical approval was obtained for the study, and all horses and personnel involved were licensed under the UK Animals (Scientific Procedures) Act.

Control blood and urine samples were collected for up to 4 days prior to the first dose. Blood samples were taken immediately before and at approximately 24, 48, 72, 96, 120, and 144 h following each dose. Additional blood samples were also taken 0.5, 1, 2, 3, 4, 6, 8, 10, 12, 14, 18, 20 h post‐first and final doses, as well as 22, 27, 31, 35, 55, 79, 103, 127, 151, 168, 175, 192, 216, 240, 264, 288, 336, 360, 384, 408, 432, 456, and 504 h post‐final dose. For the duration of the study, blood samples were taken via an intravenous catheter (Milacath) placed into the left jugular vein of the horse during the two intensive sampling periods, then removed, and in between these time points, blood was taken by direct venepuncture. Blood samples were collected in lithium heparin tubes and were centrifuged to separate plasma immediately after the collection. All naturally voided urine samples were collected as free catch samples into a lined jug for the first 24 h post‐first and final dose (the first sample collected between 15 and 35 min post‐dose, with up to 16 samples collected in the first 24 h post‐final dose) as well as six times on day 2 post‐final dose, twice daily on days 3–8, and once daily on days 9–19, with a final sample taken on day 21. The final four time points were not collected for 4 of the 6 horses, with sample collection ending on day 16 for both matrices. Urine and plasma administration samples were stored at −20°C prior to analysis.

### 2.2 Chemicals and Reagents

Ammonium acetate and potassium dihydrogen orthophosphate were procured from Sigma‐Aldrich (Dorset, UK). Methanol (MeOH), acetonitrile (ACN), ethyl acetate, sodium hydroxide, glacial acetic acid, and hexane were obtained from Fisher Scientific Ltd. (Leicestershire, UK). Reagent‐grade water (RG H_2_O) was purified by a Triple Red Ultra Clear Duo Water system (Triple Red Ltd., Buckinghamshire, UK). Chromasolv MeOH was purchased from Honeywell Research Chemicals (Berkshire, UK). Blank equine urine provided by the CRS (Suffolk, UK) and blank equine plasma purchased from TCS Biosciences Ltd. (Buckinghamshire, UK) were used to prepare pooled matrices for extracted blank (EB) and calibration line/quality control (QC) samples.

Omeprazole was purchased from Sigma Aldrich (Dorset, UK), and the internal standard (IS), D_3_‐omeprazole, was purchased from Toronto Research Chemical (Ontario, Canada). Stock solutions at a concentration of 1 mg/mL were prepared in MeOH and subsequently diluted to obtain spiking solutions at appropriate concentrations.

### 2.3 Sample Analysis

Administration plasma and urine samples were analyzed using a quantitative method, which had previously been validated for omeprazole using measures of linearity, intra‐ and inter‐batch precision and accuracy, specificity, selectivity, and sensitivity (adhering to internal EHSLC quantitative method validation guidelines) (Viljanto et al. 2018).

#### 2.3.1 Plasma

Analysis was performed over a total of six batches with the calibration range of 0.025–20 ng/mL (lower limit of quantification (LLOQ of 0.025 ng/mL)). EB samples (with and without added IS), calibration, and QC samples (low at 0.075 ng/mL, medium at 10 ng/mL and high at 17 ng/mL) were prepared in duplicate for each sample analysis batch.

1 mL of plasma was aliquoted, and 25 μL of D_3_‐omeprazole (100 ng/mL) was added to each sample. The plasma proteins were precipitated by the addition of 0.5 mL of ACN and subsequently diluted with 7 mL of aqueous phosphate buffer (1 M, pH 6.8). Samples then underwent solid‐phase extraction (SPE) using pre‐conditioned (2 mL of MeOH followed by 2 mL of RG H_2_O) Bond Elut Nexus cartridges (60 mg, 3 mL). Following the loading of samples, the cartridges were washed with 1 mL of RG H_2_O prior to elution with 1 mL of MeOH:ACN (60:40, *v:v*). Eluates were then evaporated to dryness in ambient temperature under oxygen‐free nitrogen and reconstituted in 400 μL each of Chromasolv MeOH and aqueous acetate buffer (10 mM, pH 6.8).

#### 2.3.2 Urine

Analysis was performed over a total of five batches with the calibration range of 0.1–20 ng/mL (LLOQ of 0.1 ng/mL). Matrix EB samples (with and without added IS), calibration, and QC samples (low at 0.75 ng/mL, medium at 10 ng/mL, and high at 17 ng/mL) were prepared in duplicate for each sample analysis batch.

1 mL of urine was aliquoted, and 50 μL of D_3_‐omeprazole (100 ng/mL) was added to each sample. The urine was diluted with 1 mL of aqueous acetate buffer (0.1 M, pH 9.0) prior to centrifugation for 15 min. Samples then underwent SPE using preconditioned (2 mL of MeOH followed by 2 mL of RG H_2_O) Bond Elut Nexus cartridges (60 mg, 3 mL). Following the loading of samples, the cartridges were washed with 2 mL of hexane prior to elution with 2 mL of MeOH:ethyl acetate (10:90, *v:v*). To the eluates, 1.5 mL of RG H_2_O was added to elicit liquid–liquid extraction. The organic layer was then transferred to a fresh tube, evaporated to dryness in ambient temperature under oxygen‐free nitrogen, and reconstituted in 400 μL each of Chromasolv MeOH and aqueous acetate buffer (10 mM, pH 6.8).

#### 2.3.3 Analytical Method

Plasma and urine sample analysis was performed using a Waters Xevo TQ‐S triple quadrupole mass spectrometer (MS) and Agilent Acquity I‐Class ultra‐performance liquid chromatography (UPLC) system. MS was operated in positive electrospray ionization mode at a capillary voltage of 3.2 kV, a source temperature of 150°C, a desolvation gas temperature of 500°C, and a desolvation gas flow of 1000 L/h. The collision gas used was argon, at a flow rate of 0.15 mL/min. The selective reaction monitoring (SRM) transitions used for omeprazole were 346.1 > 136.2 (collision energy (CE): 30 eV; quantitative transition), 346.1 > 151.1 (CE: 16 eV), and 346.1 > 198.1 (CE: 8 eV) at a cone voltage of 4 V. For the IS, D_3_‐omeprazole, the SRM transition used was 349.1 > 198.1 (CE: 8 eV, cone voltage 4 V).

Chromatographic separation was achieved using a Waters Acquity UPLC HSS T3 column (2.1 × 100 mm, 1.8 μm) with the column temperature set to 60 °C and using acetate buffer in Chromasolv MeOH (10 mM, pH 6.8) and aqueous acetate buffer (10 mM, pH 6.8) as mobile phases. The initial gradient conditions were 20% organic, which was held for 0.5 min, increased linearly to 99% at 5.5 min, and held for 1 min before restoring initial conditions. A flow rate of 0.4 mL/min and an injection volume of 2 μL were used. The weak wash was 20% Chromasolv MeOH in RG H_2_O, and the strong wash was Chromasolv MeOH.

### 2.4 Pharmacokinetic Analysis

**FIGURE 1:**
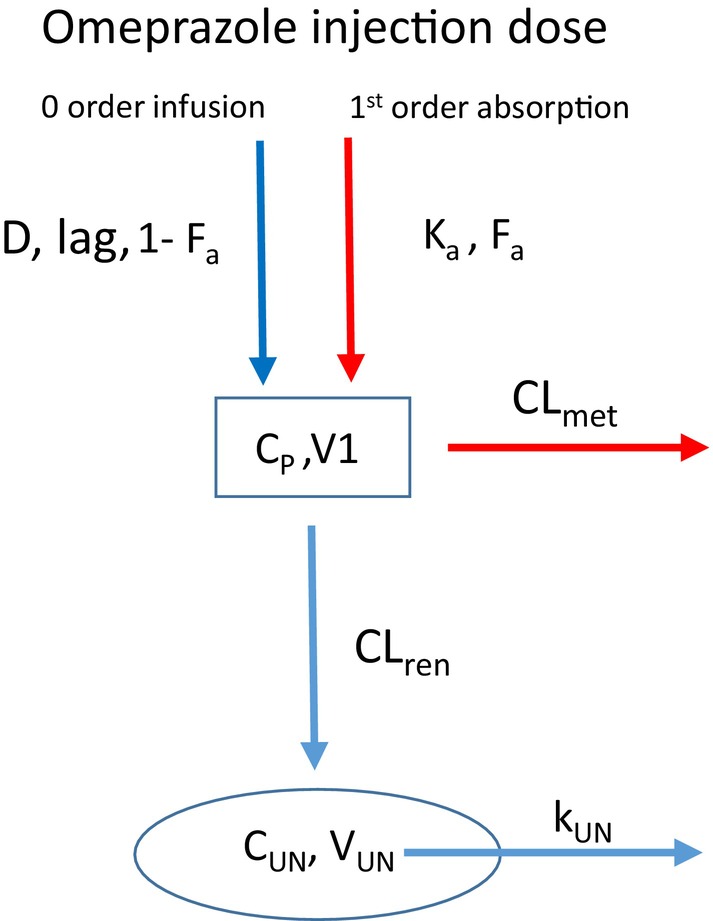
Compartmental model describing plasma and urine concentrations of omeprazole from long‐acting injection.

The compartmental model used to describe omeprazole concentrations in plasma and urine from the long‐acting omeprazole injection is shown in Figure 1 and assumes that 100% of the omeprazole injection is absorbed into the circulatory system. The model is based on a previously published model by Paine et al. (2023) that relates plasma to urine drug concentrations. In Figure 1, K_a_ and F_a_ are the first‐order absorption rate constant and fraction of dose absorbed for soluble omeprazole, respectively. In addition, D, lag and 1‐F_a_ are the infusion duration, lag time, and fraction of dose absorbed for the solid form of omeprazole, respectively. CL_met_ and CL_ren_ are the clearances for omeprazole metabolism and renal excretion from plasma. V_1_ and V_UN_ are the volumes of the central compartment and urine in the nephrons, respectively. K_UN_ is the drug elimination rate constant for the urine of nephrons. All parameters were allowed to be optimized with the exception of V_UN_, which was fixed at 0.75 mL/kg. C_P_ and C_UN_ are the plasma and urine concentrations of omeprazole, respectively. The urine production rate (UP) was determined from V_UN_ × K_UN_.

Pharmacokinetic (PK) analyses were conducted using the naive pooled algorithm within Phoenix WinNonlin 8.3 (Certara, Princeton, NJ, USA). Compartmental PK models were applied simultaneously to the plasma and urine concentration data for omeprazole. Residual error was modeled on a proportional error model. Within some horses, systematic variability in the PK was observed for the occasional dose, and therefore several models were fitted to the data. A categorical covariate for specific dose intervals within the same animal was implemented on the model parameters in a multiplicative exponential way. The model analysis started from the basic compartmental models without the covariate. Next, the covariate was applied to specific dose intervals within the same animal that appeared to have a differing shape profile. Selection of the best model was based on the lowest value of the Akaike and Bayesian Information Criteria (AIC and BIC) and visual inspection of the resulting conditional weighted residual errors. The average steady‐state plasma concentration was calculated from the dose given divided by the plasma clearance over the dosing interval. The steady‐state ratio between urine and plasma concentrations (Rss) for omeprazole was determined by dividing renal clearance (CL_ren_) by urine production rate (UP). The DTs were determined by the time post‐final dose when all horses had measured concentrations below the ISL.

## 3 Results

### 3.1 Omeprazole Pharmacokinetics

**FIGURE 2:**
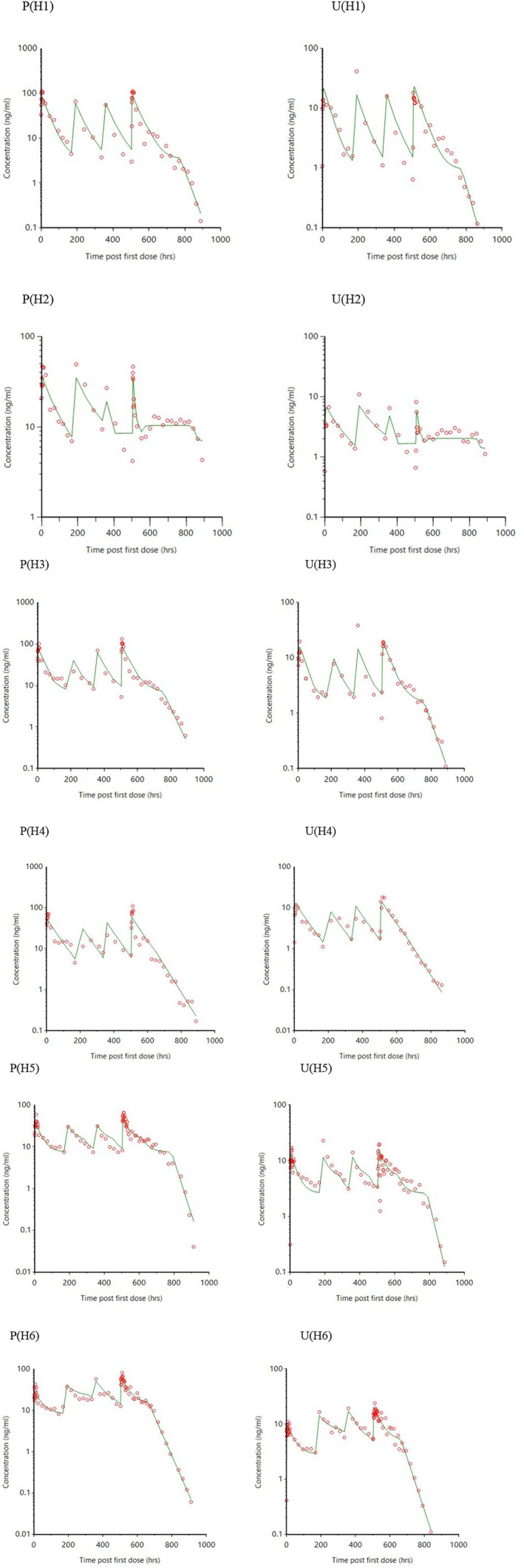
Pharmacokinetic profiles (red open circles) for omeprazole and corresponding model fits (green lines) for plasma (P) and urine (U) concentrations resulting from the oral administration of long‐acting omeprazole (4 mg/kg once per week for 4 weeks) to six horses (H).

The plasma and urine PK profiles for omeprazole resulting from intramuscular administration of a long‐acting formulation of omeprazole (6 horses) given once per week for 4 weeks are displayed in Figure 2. Plasma and urine decay curves appear to mirror one another, having complex phases of decline, and after the final dose, enter into a terminal phase of decline with a steeper slope than the preceding phase.

Table 1 shows the outputted PK parameters resulting from the model described in Figure 1. The mean metabolic and renal clearances were estimated to be 908 and 0.25 mL/h/kg (15 and 0.004 mL/min/kg) indicating that metabolism is the predominant route of elimination. The estimated period of infusion from the solid form of omeprazole ranges from 143 to 789 h, although the latter horse appears to be an exception, and for the other 5 horses, the range is 143 to 299 h. The average estimated fraction absorbed of soluble omeprazole form (F_a_) is 66%, ranging from 40% to 93%, and Rss ranged from 0.26 to 0.46.

**TABLE 1:** PK parameter values.

Parameter	H1	H2	H3	H4	H5	H6	Mean	CV%
*V*_1_ (mL/kg)	1139	351	464	608	760	375	616	44
CL_met_ (mL/h/kg)	872	690	771	1035	1162	916	908	17
*K*_*a*_ (/h)	0.02	0.01	0.02	0.02	0.03	0.02	0.02	29
*D* (h)	269	789	154	143	280	160	299	75
*F*_*a*_	0.80	0.55	0.80	0.93	0.40	0.49	0.66	29
*V*_UN_ (mL/kg)	0.75	0.75	0.75	0.75	0.75	0.75	0.75	—
CL_ren_ (mL/h/kg)	0.17	0.09	0.08	0.14	0.38	0.62	0.25	79
*K*_UN_ (/h)	0.63	0.46	0.34	0.56	1.09	1.82	0.81	62
Rss	0.36	0.26	0.31	0.33	0.46	0.45	0.36	22

Horse 2 (H2) and horse 6 (H6) showed lower exposure than expected for some of the doses i.e., for H2 and H6, exposure appears low for dose 3, and 1, respectively (Figure 2). Table 2 shows where a covariate was added to the model, and Figure 2 shows the individual horse concentration predictions, using the model estimated parameters, as green lines superimposed onto the measured concentrations (red circles) versus time graph.

**TABLE 2:** PK lag, co‐variates and residual error (RE) as CV%.

Parameter	H1	H2	H3	H4	H5	H6
Lag time (h)	No	48	94	No	No	No
Covariate *K*_*a*_	No	2.12	No	No	No	0.15
Covariate *D*	No	1.33	No	No	No	0.89
Covariate *F*_*a*_	No	−2.29	No	No	No	−0.59
RE (*P*) (%)	30	24	28	37	26	21
RE (*U*) (%)	44	33	34	27	35	21

## 4 Discussion and Conclusion

The pharmacokinetics (PK) of this long‐acting omeprazole formulation shows a complex profile with a combination of both fast and slow absorption from the injection site into the blood. This is consistent with a suspension formulation where omeprazole solubility is saturated. Therefore, a PK compartmental model was developed that has both first‐order absorption into blood for soluble omeprazole and a zero‐order infusion for solid‐phase omeprazole. For the zero‐order infusion, some horses showed a delay before infusion was initiated, and therefore a time lag was introduced into the model. Two horses showed lower exposure than expected for some of their doses, and for these cases a covariate was introduced into the model. These lower exposures may be a result of the formulation being trapped in tissue upon injection.

For 5 out of the 6 horses, the terminal phase of decline post last dose had a steeper slope than the preceding phase. This may appear unusual at first glance, but can be explained by the elimination phase transitioning from (1) systemic rate‐limiting elimination from rapidly absorbed soluble omeprazole (initial drop) to (2) much slower solubility rate‐limiting elimination from slow infusion of solid omeprazole (middle phase) back to (3) faster systemic‐limiting elimination (terminal phase) when all of the solid omeprazole has completely dissolved. Plasma concentrations were then linked to urine concentrations based on a previously published model by Paine et al. (2023).

The estimated total clearance for this long‐acting formulation of omeprazole was 15 mL/min/kg, which compares favorably with intravenous studies in horses: 12.9 mL/min/kg (Sykes, Underwood, et al. 2015) and 14.7 mL/min/kg (Jenkins et al. 1992). This supports the presumption in the model that bioavailability of omeprazole for this formulation is close to 100%. Due to the very low rate of renal clearance, the Rss is low (average 0.36), whereby concentrations of omeprazole in plasma are in fact higher than in urine. This is lower but of similar magnitude to the Rss determined by Viljanto et al. (2018) for the oral administration of omeprazole (Rss = 1).

The average steady‐state plasma concentration (C_ave,ss_) of omeprazole for the four times, once‐weekly administration of this long‐acting formulation, is 26.5 ng/mL (average for 6 horses). This is similar to C_ave,ss_ determined by Knych et al. (2017) (13 ± 6 ng/mL), overlaps with Sykes, Hewetsone, et al. (2015); Sykes, Underwood, et al. (2015) (17–87 ng/mL) but is significantly lower than Viljanto et al. (2018) (62–182 ng/mL) for the daily oral administration of 4 mg/kg omeprazole. However, in the Viljanto et al. (2018) study, plasma trough concentrations (C_min_) of omeprazole were typically less than 1 ng/mL, whereas in this study with a long‐acting formulation of omeprazole, plasma C_min_ is typically 10 ng/mL.

Interest in the novel long‐acting omeprazole product was first reported by Sykes et al. (2017) following a pilot study that suggested it suppressed acid for 7 days post a single injection. This longer action is desirable in clinical cases to prevent ulcer formation/recurrence and is greater than the reported acid suppression time for the licensed oral formulation. Subsequent clinical studies have also shown that compared to the oral formulation, the healing and reduction in lesion severity of ulcers in both equine squamous gastric disease (ESGD) and equine glandular gastric disease (EGGD) is superior with the long‐acting formulation and includes clinical cases refractory to the oral treatment (Gough, Hallowell, and Rendle 2020, 2022). This is both clinically and behaviorally desirable for the welfare of the equine patient. Practically, it may be more appealing to give an injection once per week rather than oral medication daily over a long period of time. Omeprazole acts as a quasi‐irreversible inhibitor of the parietal cell proton pump ATPase, and therefore it may be hypothesized that peak plasma concentrations (C_max_) should drive the clinical effect. However, the plasma pharmacokinetics for this long‐acting omeprazole formulation suggest that C_min_ drives the clinical effect, which would explain the superior clinical effects over oral omeprazole.

Based on the current omeprazole plasma and urine ISLs (both 1 ng/mL) and observed concentrations, the detection time for this long‐acting omeprazole formulation administered at 4 mg/kg once per week is greater than 384 h (16 days) in plasma and 384 h (16 days) in urine. Realistically, this would make treatment for horses in training with the long‐acting product challenging within the rules of racing. It would therefore most likely be used for horses outside of training, and the oral formulation would still be legitimately used during training.

Because this injectable omeprazole formulation contains compounded excipients, which do not undergo consistent testing for identity, quality, strength, purity, and stability, the results of the research described may not be reproducible for this product or, for that matter, any other long‐acting omeprazole products.

## Author Contributions

M.V. and C.H. contributed to the analytical development method, validation, and sample analysis. J.H.‐B. led the administration study and P.H. performed project management. S.W.P. coordinated the data and performed pharmacokinetic analysis. All authors contributed to the writing of the manuscript and have read and approved the final manuscript.

## Ethics Statement

The study was approved by the BHA CRS Animal Welfare and Ethics Review Board and the BHA ethics board, with the horses and personnel involved licensed under the UK's Animals (Scientific Procedures) Act.

## Conflicts of Interest

The authors declare no conflicts of interest.

## Data Availability

The data that support the findings of this study are available from the BHA. Restrictions apply to the availability of these data, which were used under license for this study. Data are available from the authors with the permission of the BHA.
